# Extreme plastomes in holoparasitic Balanophoraceae are not the norm

**DOI:** 10.1186/s12864-023-09422-1

**Published:** 2023-06-15

**Authors:** Woorin Kim, Thea Lautenschläger, Jay F. Bolin, Mathew Rees, Albertina Nzuzi, Renchao Zhou, Stefan Wanke, Matthias Jost

**Affiliations:** 1 Institut für Botanik, Technische Universität Dresden, Dresden, Germany; 2 Botanischer Garten Hamburg, Universität Hamburg, Hamburg, Germany; 3 Department of Biology, Catawba College, Salisbury, USA; 4 School of GeoSciences, University of Edinburgh, Edinburgh, UK; 5 Royal Botanic Garden, Edinburgh, UK; 6 Instituto Nacional da Biodiversidade e Conservação, Luanda, Angola; 7 State Key Laboratory of Biocontrol and Guangdong Provincial Key Laboratory of Plant Resources, Sun Yat-Sen University, Guangzhou, China; 8 Departamento de Botánica, Instituto de Biología, Universidad Nacional Autónoma de México, Mexico City, Mexico

**Keywords:** Genetic code change, Heterotrophic lifestyle, *Mat*K gene, Nucleotide compositional bias, Plastome condensation, Santalales

## Abstract

**Background:**

Balanophoraceae plastomes are known for their highly condensed and re-arranged nature alongside the most extreme nucleotide compositional bias known to date, culminating in two independent reconfigurations of their genetic code. Currently, a large portion of the Balanophoraceae diversity remains unexplored, hindering, among others, evolutionary pattern recognition. Here, we explored newly sequenced plastomes of *Sarcophyte sanguinea* and *Thonningia sanguinea*. The reconstructed plastomes were analyzed using various methods of comparative genomics based on a representative taxon sampling.

**Results:**

*Sarcophyte,* recovered sister to the other sampled Balanophoraceae s. str., has plastomes up to 50% larger than those currently published. Its gene set contains five genes lost in any other species, including *mat*K. Five *cis*-spliced introns are maintained. In contrast, the *Thonningia* plastome is similarly reduced to published Balanophoraceae and retains only a single *cis*-spliced intron. Its protein-coding genes show a more biased codon usage compared to *Sarcophyte,* with an accumulation of in-frame TAG stop codons*.* Structural plastome comparison revealed multiple, previously unknown, structural rearrangements within Balanophoraceae.

**Conclusions:**

For the “minimal plastomes” of *Thonningia*, we propose a genetic code change identical to sister genus *Balanophora*. *Sarcophyte* however differs drastically from our current understanding on Balanophoraceae plastomes. With a less-extreme nucleotide composition, there is no evidence for an altered genetic code. Using comparative genomics, we identified a hotspot for plastome reconfiguration in Balanophoraceae. Based on previously published and newly identified structural reconfigurations, we propose an updated model of evolutionary plastome trajectories for Balanophoraceae, illustrating a much greater plastome diversity than previously known.

**Supplementary Information:**

The online version contains supplementary material available at 10.1186/s12864-023-09422-1.

## Background

Holoparasitic Balanophoraceae maintain some of the most extreme plastomes known to date. Considerable structural reconfigurations alongside a highly condensed gene set have identified the family as key lineage for the study of evolutionary processes of so-called “minimal plastomes” [1–4]. Their nucleotide composition represents the most extreme reported for this organelle so far and is accompanied by two independent modifications of the underlying genetic code [1, 3].

Balanophoraceae (Santalales), consisting of 42 species from 14 genera, is broadly distributed across (sub-) tropical regions worldwide [5–7]. Relationships within the family are partially uncertain as molecular data for a large portion of the diversity are currently lacking [3, 8]. Balanophoraceae are estimated to be among the oldest parasitic angiosperm lineages (~ 110 – 115 mya) alongside Cynomoriaceae and Hydnoraceae [9]. However, the limited taxon sampling and the effect of elevated nucleotide substitution rates heavily influence such estimations. Complete plastomes of only four Balanophoraceae genera have been characterized to date, all having lost the quadripartite genome structure and displaying similarly condensed size and gene content as well as nucleotide compositional bias. The Balanophoraceae minimal plastomes range from ~ 17.3 kb – 20.9 kb in genera *Lophophytum*, *Ombrophytum*, and *Rhopalocnemis* [3, 4, 10] and from ~ 14.6 kb – 16 kb in genus *Balanophora* [1, 2]. In the latter, all *cis*-spliced introns have been lost and intergenic spacers (IGS) are drastically reduced with various partially overlapping genes [1, 2]. Balanophoraceae plastomes contain a shared set of 13 – 19 genes, comprised of a variable set of rRNAs, ribosomal proteins (*rpl* and *rps*), and protein coding genes with other functions (*acc*D, *clp*P, *ycf*1, and *ycf*2), as well as a single tRNA (*trn*E-UUC) in *Balanophora* [3]. Adenine and thymine make up to 88.4% of the plastomes of *B. reflexa* [1] and *Lophophytum leandri* [3], compared to an average of 61 – 66% A + T in autotrophic angiosperms. Additional published Balanophoraceae genera confirm this general trend towards low nucleotide complexity plastomes [2–4, 10]. This extreme bias likely resulted in two independent codon reassignments in Balanophoraceae. In *Ombrophytum* and *Lophophytum* plastomes, tryptophan is likely encoded by TGA in addition to only TGG [3], whereas TAG is indicated to additionally encode tryptophan in plastomes of *Balanophora* [1]. As a result of these reassignments, translation termination is almost exclusively realized using TAA, even in *Rhopalocnemis*, for which no altered genetic code has been observed.

**Fig. 1:**
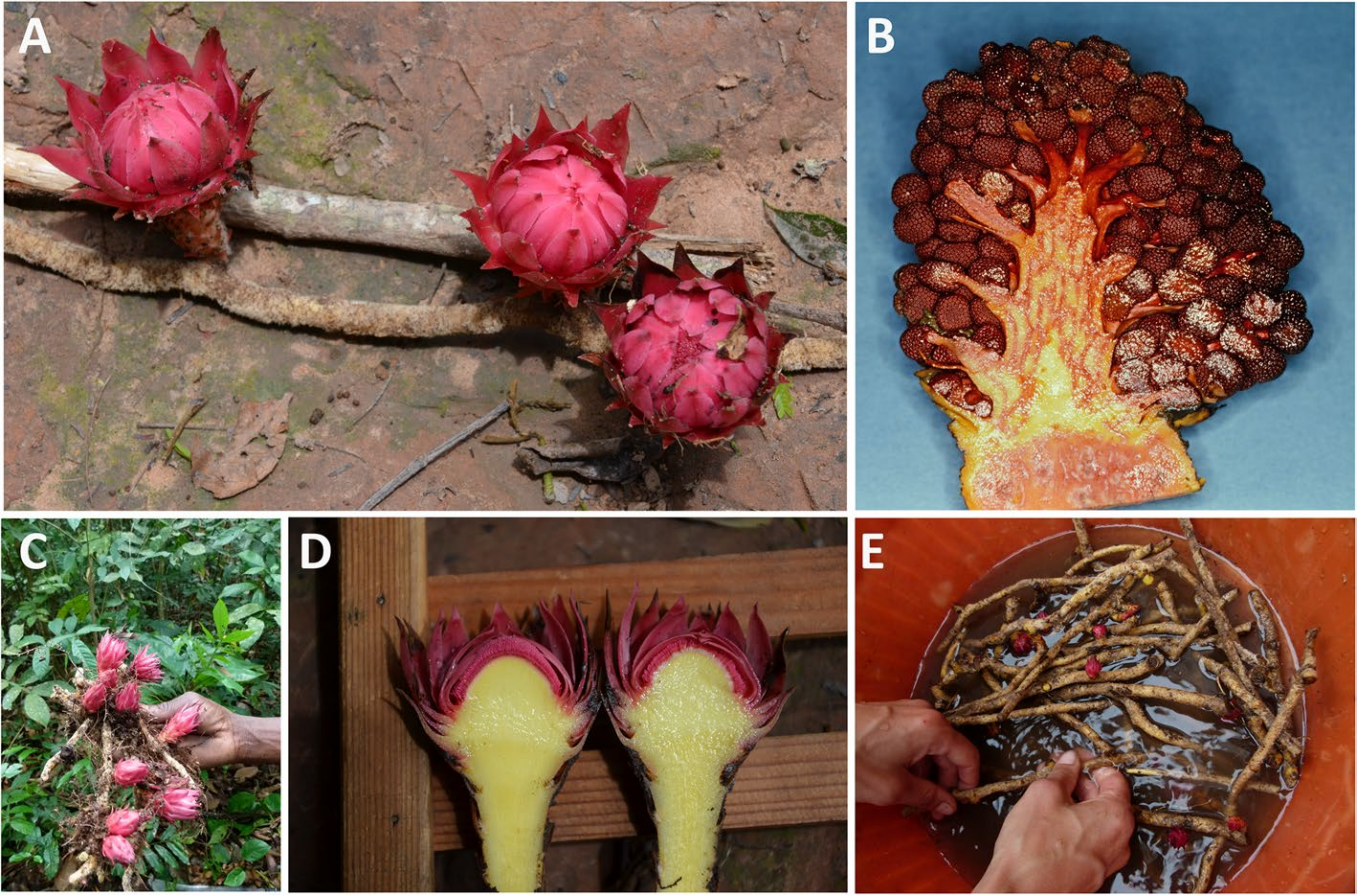
The unique morphology of *Thonningia sanguinea* (**A**, **C**—**E**) and *Sarcophyte sanguinea* (**B**). Longitudinal sections of the *Sarcophyte* (**B**) and *Thonningia* (**D**) inflorescences. Their rhizomes are commonly traded on local markets, shown here for *Thonningia* (**E**). Picture credit: Barbara Ditsch provided (**A**) and (**D**), Daniel Nickrent (**B**)

All previous studies paint a picture of Balanophoraceae plastomes that are exceptional in most categories. However, these conclusions are based on only ~ 30% of the known genus diversity. Here, we report the newly sequenced and assembled plastomes of two additional Balanophoraceae genera, *Sarcophyte sanguinea* Sparrm. and *Thonningia sanguinea* Vahl (Fig. 1). We also assessed intraspecific variation by using two and three biological replicates from different origins, respectively. Using comparative approaches, we explore gene and intron losses, structural variation as well as nucleotide compositional bias and changes in genetic code. Based on a fully resolved phylogenetic hypothesis, we propose an expanded model for possible evolutionary trajectories in Balanophoraceae.

## Methods

### Plant materials, DNA extraction and sequencing

Genomic DNAs of *Thonningia sanguinea* (3 accessions) and *Sarcophyte sanguinea* (2 accessions) were isolated from silica gel dried plant material and sequenced via next generation sequencing. Two specimens of *Thonningia sanguinea* were collected in Angola with permits granted by the Instituto Nacional da Biodiversidade e Áreas de Conservação (INBAC, Ministério do Ambiente, N.145/INBAC.MINAMB/2013) and the Governo Provincial de Uíge (52/GD/IP-UNIKIVIY2022) and deposited in the herbarium of the Royal Botanic Garden Edinburgh (Rees 90 and Rees 123, identified by Mathew Rees). The third biological replicate corresponds to a herbarium specimen (Herbarium Dresdense, DR077682, identified by Thea Lautenschläger). Both studied accessions of *Sarcophyte sanguinea* correspond to herbarium specimens, one in the collection of the Herbarium Dresdense (DR077681, identified by Jay F. Bolin) and the second in the collection of the East African Herbarium (Matheka K. 202101, identified by Kennedy Wambua Matheka). DNA extraction followed the protocol of Doyle and Doyle [11], modified to include RNAase A (Thermo Scientific, Waltham, MA, USA) treatment (10 mg/ml). DNA concentration and quality were measured using a Qubit 3 Fluorometer (Thermofisher Scientific, Waltham, MA, USA). Sequencing was carried out on an Illumina NovaSeq 6000 system, as 150 bp paired-end reads for 300 cycles, aiming for about 80 million reads per sample.

### Data mining from public repositories

For comparative analysis and phylogenetic tree reconstruction, plastomes of Balanophoraceae as well as selected outgroup lineages were mined from the NCBI nucleotide database. The complete list of plastomes used in this study can be found in the Additional file 1. We annotated the plastomes of *Balanophora harlandii* (MN414177; [2]) and *Balanophora fungosa var. globosa* (MN414176; [2]) following the methods described below, as the published plastomes did not contain any annotations.

### Raw data assembly and plastid genome reconstruction

Raw read data were trimmed for sequencing adapters and quality using BBDuk v. 1.0 [12]. Trimmed read data were de novo assembled using two different methods and cross-checked. De novo assembly after filtering for plastid-like reads was done using the GetOrganelle v. 1.7.4.1 [13] pipeline, with standard settings. De novo assembly on all sequence raw data was done using CLC Genomics Workbench (v. 11.0, Qiagen, MD, USA), allowing for automatic calculation of optimal word and bubble sizes for each sample. For each assembly, read-mappings were created using CLC Genomics Workbench (v. 11.0, Qiagen, MD, USA) and assessed using Tablet v. 1.21.02.08 [14].

Gene annotation of newly sequenced plastomes was done in Geneious (v. 11.1.5, Biomatters, Ltd., New Zealand) using the published plastomes of Balanophoraceae [1–3, 10] and autotrophic *Erythropalum scandens* (NC_036759) [15] as references. Annotations were manually inspected and adjusted where necessary. Additionally, open reading frames (ORFs) of protein coding genes were investigated using HMMER [16] to detect homologs using various protein sequence databases. Sequence and secondary structure of tRNAs were predicted using tRNAscan-SE v. 2.0 [17]. Plastomes were visualized using OGDraw [18].

Relative synonymous codon usage (RSCU) [19] of the Balanophoraceae protein-coding genes was calculated using MEGA11 [20].

### Phylogenetic analysis

Single gene alignments for 19 protein-coding genes and 4 ribosomal RNAs were created using MAFFT v. 7.450 [21, 22] and manually adjusted in PhyDE (v 0.9971) and AliView [23]. We excluded *rrn*5 from the analysis, considering its often highly divergent sequence and uncertain functionality. All other genes were concatenated using SequenceMatrix v. 1.8 [24]. PartitionFinder2 [25] was used to identify the best-fitting nucleotide substitution model (GTR + I + G). Phylogenetic inferences were estimated with RAxML v. 8.2.12 [26], implemented on Cipres Science Gate [27], using a gene partitioning approach. Boostrap support (BS) values are based on 1,000 replicates. Tree files were visualized using TreeGraph 2 [28].

## Results

### The plastomes of *Sarcophyte* and *Thonningia*

**Fig. 2:**
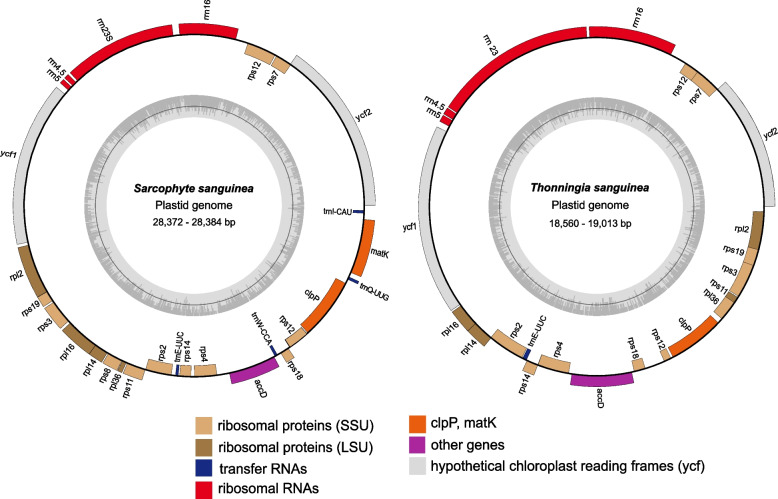
The reconstruction of a less condensed plastome in Balanophoraceae. Plastome lengths, given as a range, correspond to two biological replicates of *Sarcophyte sanguinea* and three for *Thonningia sanguinea*. Genes are color-coded according to functional groups. Genes inside the outer circle are transcribed clockwise, and genes on the outside are transcribed counter-clockwise. The inner grey histogram represents the GC content, with the middle line highlighting 50% GC at each bin

Plastome reconstruction resulted in complete, circular molecules for both accessions of *Sarcophyte sanguinea* and the three accessions of *Thonningia sanguinea*, all lacking a quadripartite genome structure. The plastomes of *Sarcophyte* (28,372 – 28,384 bp) are ~ 10 kb larger in size compared to those of *Thonningia* (18,560 – 19,013 bp) and exhibit a slightly larger nucleotide compositional bias (19.1% GC versus 20.4 – 21.2% GC, respectively, Additional file 1). Both *Sarcophyte* plastomes contain an identical 27-gene set, comprised of 19 protein coding genes (*acc*D, *clp*P, *mat*K, *rpl*, *rps*, and *ycf*), four rRNA genes, as well as four tRNAs (*trn*E, *trn*I, *trn*Q, and *trn*W) (Fig. 2 and Additional file 2). Four genes are found containing *cis*-spliced introns, namely *clp*P (2), *rpl*2 (1), *rpl*16 (1), and *rps*12 (1), with the latter additionally containing a *trans*-spliced intron (Additional file 1). Secondary structure prediction shows typical cloverleaf folds for all identified tRNAs, along with the presentation of the correct anticodon, as predicted by sequence similarity, except for *trn*W. Here, an UUA anticodon is predicted instead of CCA. Gene order and orientation is identical among the two plastomes of *Sarcophyte*, accompanied by a low degree of nucleotide diversity. Noteworthy is an indel in a mononucleotide repeat at the *rpl*2 3’-end, which is supported by the readmappings. The introduced frameshift leads to a gene length increase (10 bp) in accession S01, resulting in a partial overlap (4 bp) with the adjacent *rps*19 gene.

The plastomes of the three *Thonningia sanguinea* accessions share an identical 22-gene set, consisting of 17 protein-coding genes (*acc*D, *clp*P, *rpl*, *rps*, and *ycf*), four rRNA genes, and a single tRNA (*trn*E). Two introns were identified: a *cis*-spliced intron in *clp*P and a *trans*-spliced intron in *rps*12 (Fig. 2 and Additional file 2). Gene annotation using the plant plastid code revealed frequent inclusion of premature stop codons (exclusively TAG) in twelve out of 17 protein-coding genes. No TAG stop codons were identified at any 3’-terminus in *Thonningia*. Nucleotide diversity between accessions T02 and T03 was lower compared with T01; however, gene order and orientation is synonymous among all three. A noteworthy structural distinction unique to accession T02 is the lack of gene overlap for the *rpl*36 5’-end with the adjacent *rpl*2 3’-end, which is present in both T01 and T03 (4 bp). The gene length of the T01 *rpl*16 gene is much shorter (303 bp) compared to the other two accessions (both 405 bp) due to a point mutation altering the start codon. The next in-frame canonical start codon was chosen for the *rpl*16 annotation of T01.

### Gene and intron losses and structural variations in the Balanophoraceae plastomes

**Fig. 3:**
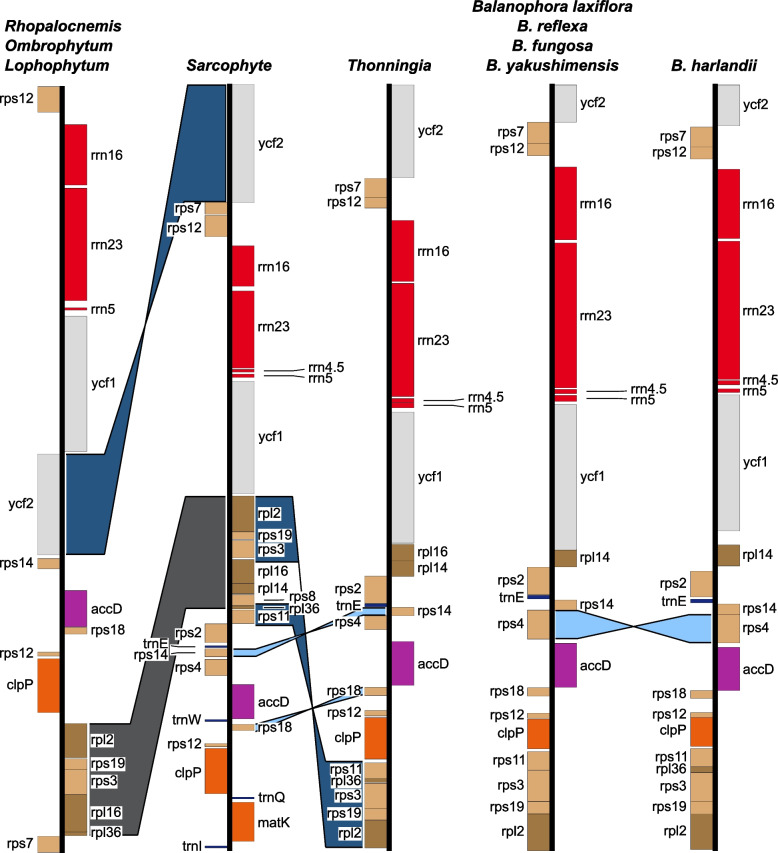
Linear comparison of Balanophoraceae plastomes highlights events of reconfigurations. Genome lengths are not to scale. Differences in gene order and orientation are highlighted by colored areas: translocations in dark gray, inversions in light blue and translocations + inversions in dark blue. Genes are color-coded based on functional groups used in Fig. 2. *Rhopalocnemis* was chosen to display the plastomes of *Ombrophytum * and *Lophophytum*, which additionally contain the *rps*4 gene in between *acc*D and *rps*14 and are both missing the *rpl*16 gene. *Lophophytum* lacks *rpl*36. The *Balanophora laxiflora* plastome was selected as representative for the plastomes of *B. reflexa*, *B. fungosa*, and *B. yakushimensis.* Plastome gene content in *B. fungosa* differs from the other aforementioned *Balanophora* species in the presence of an *rpl*36 gene copy in between the *rps*3 and *rps*11 genes. Furthermore, both *B. fungosa* and *B. yakushimensis* differ in the absence of *ycf*2 compared to the other species

Annotation of published Balanophoraceae plastomes using gene features of *Sarcophyte* allowed for the identification of *rrn*5 genes (112 – 125 bp) and *rrn*5 gene fragments (58 – 105 bp) in all accessions. Additionally, we found and annotated an *rpl*36 gene copy in *B. harlandii* (Fig. 3, Additional file 2). This annotation is further supported by HMMER prediction and contains a complete ORF. All Balanophoraceae plastomes lack a quadripartite plastome structure and no larger repeat regions (> 30 bp) have been identified. Genome sizes range from ~ 28.4 kb in *Sarcophyte* to ~ 14.6 kb in *Balanophora yakushimensis* (Additional file 1). The nucleotide compositional bias heavily favors A and T, culminating in 11.6% GC in both *B. reflexa* and *Lophophytum* (Additional file 1). The highest GC content was identified in the plastomes of *Thonningia* (20.4 – 21.2% GC).

Balanophoraceae plastomes display varying gene and intron contents as well as changes in gene order and orientation. In the plastomes of *Sarcophyte*, we identified five genes that are missing in all other Balanophoraceae, namely *mat*K, *rps*8 as well as tRNAs I, Q, and W (Additional file 2). In both *B. yakushimensis* and *B. fungosa*, the *ycf*2 gene has been lost. *Balanophora laxiflora*, *B. reflexa*, and *B. yakushimensis* as well as *Lophophytum* share the loss of *rpl*36 (Fig. 3). Furthermore, *Lophophytum*, *Ombrophytum*, and *Rhopalocnemis* are missing *rpl*14, *rps*2, *rps*11, and *rrn*4.5 compared to the other Balanophoraceae studied here. Lastly, *rps*4 is missing from the *Rhopalocnemis* plastomes (Fig. 3 and Additional file 2). In Balanophoraceae, at least five independent intron losses have been identified. *Balanophora* is yet the only known genus of the family having lost all *cis*-spliced introns. *Thonningia* has lost the *cis*-spliced *rps*12 and *rpl*2 introns. The *clp*P gene, containing two *cis*-splicing introns in *Sarcophyte*, *Lophophytum*, *Ombrophytum*, and *Rhopalocnemis*, lacks intron 2 in *Thonningia* (Additional file 2). We identified a similar pattern of intron loss for *rpl*16. The gene is missing in *Balanophora*, *Lophopytum*, and *Ombrophytum*, present but without intron in *Thonningia* and contains a single *cis*-spliced intron in *Rhopalocnemis* and *Sarcophyte* (Additional file 2). The only intron shared among all Balanophoraceae plastomes is the trans-spliced intron of *rps*12.

Multiple events of structural discordance can be identified among the Balanophoraceae plastomes. *Balanophora harlandii* is characterized by the only inversion of the *rps*4 gene yet (Fig. 3). *Balanophora* and *Thonningia* share inversions of both *rps*14 and *rps*18, whereas *ycf*2 is inverted in *Lophophytum*, *Ombrophytum*, and *Rhopalocnemis*. Compared to the plastomes of *Sarcophyte*, the *rpl*2 – *rpl*36 (~ 2060—2800 bp) region in *Lophophytum*, *Ombrophytum*, and *Rhopalocnemis* is translocated (Fig. 3). The almost identical region, *rpl*2 – *rps*11 (~ 2000—2200 bp), is translocated and inverted in *Balanophora* and *Thonningia*, although excluding *rpl*14 and *rpl*16.

Considering the plant plastid code, internal TAG stop codons, as identified in the majority of *Thonningia* protein-coding genes, have additionally been found in genus *Balanophora*. Internal TGA stop codons, on the other hand, are recognized in *Lophophytum* and *Ombrophytum*.

### Phylogenetic relationships within Balanophoraceae

**Fig. 4:**
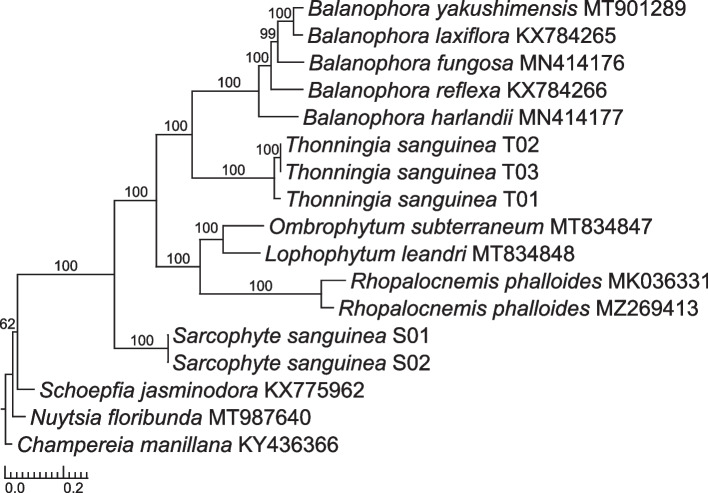
*Sarcophyte* recovered as sister to remaining Balanophoraceae. Maximum likelihood tree reconstruction based on the concatenated 22-plastid gene matrix shown as phylogram. Support values are based on 1,000 BS replicates. *Schoepfia* (Schoepfiaceae), *Nuytsia* (Loranthaceae), and *Champereia* (Opiliaceae) were used as outgroups, following Nickrent et al. [29]. *Champereia* was used for rooting

Phylogenetic analysis based on the concatenated 22-plastid gene set reveals the monophyly of Balanophoraceae, with *Sarcophyte* as the first diverged genus among the sampled genera of this family (BS 100, Fig. 4). *Lophophytum, Ombrophytum* and *Rhopalocnemis* are resolved as a monophyletic group, and place sister to a clade of *Thonningia* and *Balanophora* (BS 100 for both groups, Fig. 4). Within genus *Balanophora*, *B. harlandii* diverged first (BS 100). The branches leading to all five genera of Balanophoraceae are considerably longer than those leading to the outgroups, with the longest branch leading to *Rhopalocnemis*.

### Plastome structural changes among Balanophoraceae and close relatives

To determine the extent of plastome reconfiguration in Balanophoraceae, we compared representative plastomes of an autotrophic Santalales (*Eryhtropalum*) [15] and hemi-parasitic lineages (Loranthaceae, Opiliaceae, and Schoepfiaceae, Additional file 1). Due to identical gene order and orientation in the selected outgroup taxa, we chose closely related *Nuytsi*a (NC_058869) [30] for pairwise comparisons with Balanophoraceae. For better visualization, we created three versions of the *Nuytsia* plastome, containing only the gene content present in a) *Sarcophyte*, b) *Balanophora* and *Thonningia*, and c) *Lophophytum*, *Ombrophytum*, and *Rhopalocnemis* (Additional file 3). The pairwise comparisons revealed two translocations combined with inversions between *Sarcophyte* and *Nuytsia* (*mat*K-*trn*Q and *rps*11-*rpl*2). The gene order of *Thonningia* and *Balanophora* can be explained by two independent inversions (*rps*14, *rps*18) along with a single translocation + inversion (*rpl*14-*rpl*16). Lastly, the plastome structure of *Lophophytum*, *Ombrophytum*, and *Rhopalocnemis* differs in an inversion (*rpl*36-*rpl*2) and a translocation combined with an inversion (*ycf*2) from the *Nuytsia* plastome (Additional file 3).

### Relative synonymous codon usage analysis in Balanophoraceae

To illuminate potential patterns of bias in synonymous codon usage, we estimated the RSCU for the protein-coding genes of the Balanophoraceae plastomes. In all accessions, A/T-rich codons are greatly preferred to codons composed of C/G. Several codons are not used at all, mostly in *Balanophora*, followed by *Ombrophytum*, *Lophophytum*, and *Rhopalocnemis* (Additional file 4). Noteworthy is that the sole codon for tryptophan (UGG) is not being used in one accession of *Rhopalocnemis* (MZ269413) [4] and several *Balanophora* species. *Thonningia* and *Sarcophyte* show a less extreme, but still highly biased RSCU. Little differences in RSCU are identified among the different accessions of *Thonningia* and *Sarcophyte*, as well as within *Balanophora*.

Out of the three canonical stop codons, TAA is by far the most frequently used in the protein-coding genes of Balanophoraceae, with the exception of *Thonningia* and *Ombrophytum*, which show TGA and TAG instead, respectively (Additional file 4).

## Discussion

The Balanophoraceae plastomes published to date are considered some of the most exceptional with genome sizes of only ~ 15 – 21 kb, the most extreme nucleotide compositional bias (up to 88.4% AT) and the only two described modifications of the plant plastid translational code among land plants [1–3]. The newly sequenced plastomes of *Thonningia* are no exception to this. Assembled genomes of all three *Thonningia* accessions are to be considered “minimal plastomes”, with lengths ranging from ~ 18.6 – 19 kb and an A/T content of ~ 80% (Additional file 1). Gene annotation and codon analysis indicate an alteration of its genetic code, identical to that of its sister genus *Balanophora* (Additional file 4). In the latter, transcriptome analysis confirmed the change of a terminal codon (TAG) to a tryptophan codon, resulting in translation extension rather than termination [1].

The newly assembled plastomes of *Sarcophyte*, on the other hand, are distinct from other Balanophoraceae in various aspects. With genome sizes of ~ 28 kb, they are roughly 33 – 50% larger than their family members (Additional file 1) and classify in the upper size range of what can be considered a minimal plastome, comparable to various holoparasitic Hydnoraceae (~ 28 kb) [31] and certain species of holo-mycoheterotrophic *Epipogium* (~ 30 kb) [32]. Tree reconstruction recovered *Sarcophyte* sister to the remaining Balanophoraceae, comprised of the clades *Balanophora* + *Thonningia* and *Ombrophytum* + *Lophophytum* + *Rhopalocnemis* (Fig. 4). This result is congruent with the reconstructions estimated by Chen et al. [2] and Ceriotti et al. [3], although based on a different taxon sampling. The recovery of *Sarcophyte* as sister to all other sampled Balanophoraceae in our study (BS 100, Fig. 4) is however incongruent with its placement recovered by Su et al. [8]. In their study, *Sarcophyte* places sister to *Lophophytum* + *Ombrophytum* and *Corynaea* + *Helosis* (the latter two not included in our study) with low bootstrap support.

### Dynamic gene and intron losses in Balanophoraceae plastomes

**Fig. 5:**
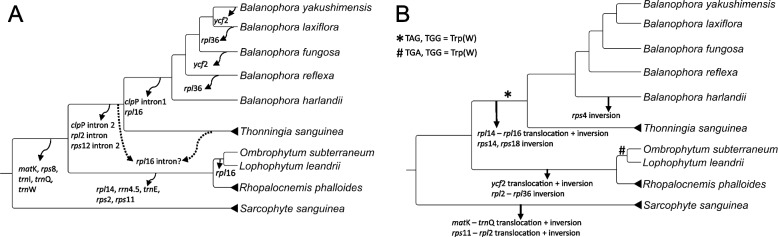
Dynamic evolution of plastome structures in Balanophoraceae. Gene and intron losses (**A**) and identified structural rearrangements (**B**) are mapped to the phylogenetic hypothesis. Structural rearrangements are in relation to the gene order and orientation identified in closely-related outgroup plastomes (Additional file 3). Two independent modifications of the plant plastid code are highlighted at their hypothesized position (* and #)

Mapping individual events of gene and intron loss on the Balanophoraceae phylogeny reveals a highly dynamic picture of microstructural evolution (Fig. 5), in line with many other heterotrophic lineages [33–35]. Sister to the remaining Balanophoraceae, *Sarcophyte* maintains the largest plastid gene set (27, Additional file 2) and shows no intron loss in its plastid genes compared to the autotrophic outgroup (*Erythropalum*, NC_036759). Among the five genes uniquely retained in *Sarcophyte* are three tRNAs (I, Q, and W) and a protein-coding gene encoding for the maturase (*mat*K, Additional file 2). The majority of additional gene losses are attributed to the branch leading to *Ombrophytum*, *Lophophytum*, and *Rhopalocnemis*, with a total of five (*rpl*14, *rps*2, *rps*11, *rrn*4.5, and *trn*E) (Fig. 5). No further genes have been lost on the branch leading to *Balanophora* and *Thonningia*. Here, plastome condensation mostly concerns the loss of group II introns. It is uncertain, whether the *rpl*16 intron was lost prior to the complete gene loss in *Balanophora* (Fig. 5). In Balanophoraceae, we identified three genes, which have each been independently lost twice. Namely, *rpl*16 in *Lophophytum* + *Ombrophytum* and *Balanophora*, *rpl*36 in *B. reflexa* and *B. laxiflora*, and *ycf*2 in *B. fungosa* and *B. yakushimensis* (Fig. 5). All retained *cis*-spliced introns in the Balanophoraceae plastomes (Additional file 2) belong to group II [36, 37] and require a maturase for mRNA splicing prior to translation. *Mat*K is the only plastid-encoded maturase and therefore considered essential for protein synthesis of group II intron-containing genes [38], with the exception of the *clp*P intron 2 [39]. Considering this, only *Sarcophyte* (presence of *mat*K and group II introns) and *Balanophora* (loss of all *cis*-spliced introns) could facilitate translation for all retained protein-coding genes. However, it seems unlikely that multiple intron-containing genes could be retained (and evolving under purifying selection; [3] in *mat*K-lacking genera without an alternative means of splicing. A plausible solution is the import of a nuclear-encoded *mat*K-like protein from the cytosol. In *Physcomitrella* such a protein has been shown to splice *clp*P pre-mRNA [40]. Complementary transcriptome data are highly desirable as additional evidence to verify successful splicing of intron-containing genes in Balanophoraceae.

All protein-coding genes in *Thonningia* and *Sarcophyte* are assumed functional based on their intact ORFs. Due to extreme sequence divergence, no further comments on the functionality of the newly identified *rrn*5 genes in the published plastomes can be made. Secondary structure prediction estimates typical cloverleaf folds for all annotated tRNAs and displays the correct anticodons as predicted by sequence similarity. *Trn*W of *Sarcophyte* is the only exception to this; it displays TTA (a stop codon) instead of the expected CCA anticodon. Tryptophan, with the exception of *Balanophora*, *Lophophytum*, *Ombrophytum*, and *Thonningia*, is encoded by only a single codon.

### Comparative analysis on genetic code reassignment, RSCU and tRNA loss within Balanophoraceae

Codon analysis of *Sarcophyte*, unearths various differences to most of the extremes observed in the family [1–3]. Similar to *Rhopalocnemis*, canonical stop codons are solely found at terminal position of the protein-coding genes. There is no evidence for *Sarcophyte* to carry any genetic code reassignments, which independently occurred twice in other groups of Balanophoraceae (Fig. 5) [1, 3]. Nevertheless, both *Sarcophyte* and *Rhopalocnemis* almost exclusively show use of the TAA stop codon (Additional file 4), similar to those Balanophoraceae plastomes with genetic code reassignments [1, 3]. RSCU analysis signals a correlation between overall plastome A/T content and codon usage bias. With the highest G/C content in Balanophoraceae, *Sarcophyte* and *Thonningia* (19.1 and 21.2% G/C, respectively) generally use a more diverse set of codons, resulting in RSCU values not as extreme as their relatives (Additional file 4). This lower bias in *Sarcophyte* and *Thonningia* can be best explained by their nucleotide composition due to neutral mutation/drift rather than selection efficiency in codon-usage [1, 41, 42]. In short, our data provide compelling evidence for a reassignment of genetic code in *Thonningia*, identical to the one identified in *Balanophora* (Fig. 5).

Codons corresponding to tRNAs retained in the plastomes of *Sarcophyte*, *Thonningia*, and *Balanophora* show no remarkable differences in their use compared to plastomes from which they are missing (Additional file 4). Considering that none of the retained tRNA gene sets are sufficient for protein synthesis, the import of cytosolic, plastid-targeted tRNAs likely exists in Balanophoraceae, in line with many other heterotrophic lineages. It is hypothesized for such an import mechanism to be able to also compensate for the presumed recent pseudogenization of *trn*E in *Balanophora* [3], as well as for the likely pseudogenized *trn*W in *Sarcophyte*. Retention of *trn*E, despite its pseudogene-like character, is presumed due to its additional function in heme biosynthesis [43–45]. Whether the same can be called for *trn*W in *Sarcophyte* is uncertain. However, in the plastome of *Prosopanche americana*, a *trn*W pseudogene-like copy with similarly altered anticodon still persists [46]. It could be hypothesized that the tRNAs retained in the Balanophoraceae plastomes may play less or no roles in protein synthesis anymore.

### Likely evolutionary trajectories of rearrangements in Balanophoraceae plastomes

Plastome structure, generally highly conserved [47], is often altered in holo-heterotrophic plants [31, 32, 35, 48–50]. Understanding the evolutionary direction of structural rearrangements is essential to study underlying mechanisms. In Balanophoraceae plastomes, we identified multiple independent events of structural reconfigurations (Fig. 3). In combination with an improved taxon sampling compared to a previous study [3] and including closely-related outgroups, we propose an updated model for structural evolution. Considering the identical plastome structures among autotrophic *Erythropalum* and hemi-parasitic *Champeria*, *Nuytsia*, and *Schoepfia* (Santalales outgroups)*,* likely representing the ancestral state, seven independent events of rearrangement have happened in Balanophoraceae (Additional file 3). Based on these rearrangements, Balanophoraceae can be subdivided into three monophyletic groups, which share identical gene order and orientation among them: A) *Sarcophyte*, B) *Thonningia* and *Balanophora* (with the exception of an inversion unique to *B. harlandii* (*rps*4, Figs. 2 and 4)), and C) *Ombrophytum*, *Lophophytum*, and *Rhopalocnemis* (Fig. 5, Additional file 3). The plastome structure of *Sarcophyte* can be explained by two events of sequence translocation and inversion. Three steps are required to explain the plastomes of *Thonningia* and *Balanophora*, one inversion and one translocation + inversion. Lastly, two steps are required for the conversion of the outgroup plastome structure to that present in *Ombrophytum*, *Lophophytum*, and *Rhopalocnemis* (Fig. 5, Additional file 3). None of the abovementioned rearrangements are shared among all Balanophoraceae, therefore having to be considered independent events. Noteworthy however is the plastome region containing the bulk of the retained ribosomal protein-coding genes (*rpl*, *rps*). Parts of this region are affected by three different rearrangement events (Fig. 3 and Additional file 3), identifying it as hotspot for plastome reconfiguration in Balanophoraceae.

Our results on plastome diversity, genetic code change and the highly dynamic nature of gene and intron losses in combination with their age identify holoparasitic Balanophoraceae as pivotal to study the general evolutionary processes acting on minimal plastomes (such as condensation), in line with mycoheterotrophic Burmanniaceae [51, 52], Orchidaceae [53], and Thismiaceae [54, 55]. Derived models will allow for estimations on putative past and future evolutionary trajectories and will further our understanding of universal aspects of plastome evolution [35]. Confident model establishment however requires more data than currently available, which is especially true for Balanophoraceae. Our study highlights, that the available data for a snippet of the known diversity of a lineage are not necessarily representative for it as a whole. Screening the NCBI database (https://www.ncbi.nlm.nih.gov/) highlights data deficiency for many holoparasitic lineages, as for only half of the approximately 40 genera of holoparasitic plants, a complete plastome reconstruction exists to date.

## Conclusions

This study compliments and expands our knowledge on plastome evolution in Balanophoraceae and holo-heterotrophic plants as a whole and highlights, for the first time, less extreme and less condensed plastomes within the family. These results indicate that Balanophoraceae plastomes show a much greater diversity than previously known and it is essential to increase sampling efforts for currently missing genera and species. Based on our results, we propose Balanophoraceae as an ideal holoparasitic lineage to study the processes of minimal plastome evolution.

## Supplementary Information

**Additional file 1.** 

**Additional file 2.** 

**Additional file 3.** 

**Additional file 4.** 

## Data Availability

The newly assembled plastomes of *Sarcophyte* and *Thonningia* are available in the NCBI nucleotide database under accession numbers OQ810027—OQ810031.
