# Supplementary material for: Extreme plastomes in holoparasitic Balanophoraceae are not the norm
Source: BMC Genomics. 2023 Jun 15;24:330. doi: 10.1186/s12864-023-09422-1 (PMC10268348; doi:10.1186/s12864-023-09422-1)
Supplement: Supplementary file 3 — Additional file 3. [file 12864_2023_9422_MOESM3_ESM.pdf]

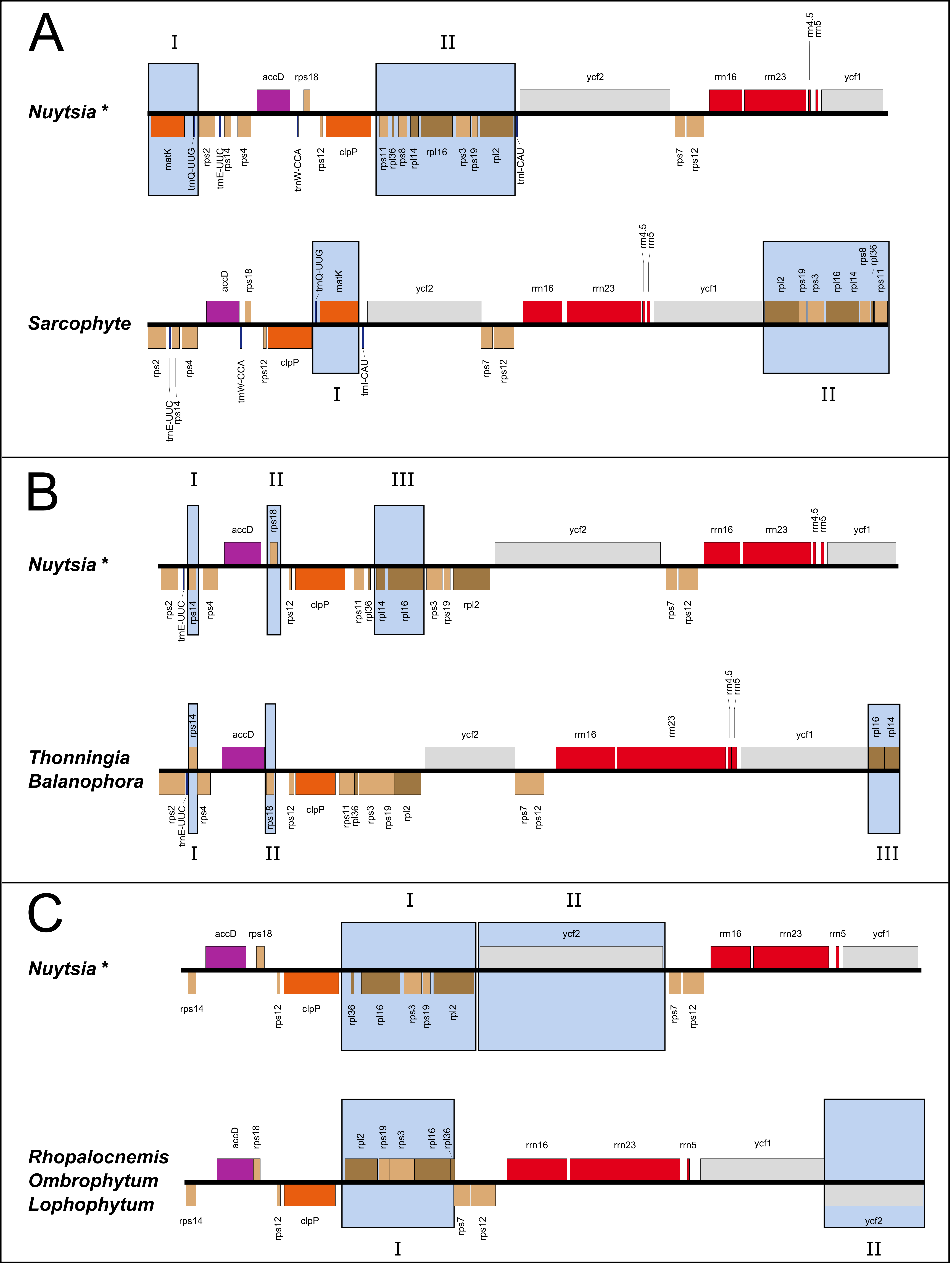

Additional file 3. Pairwise, linear comparison of Balanophoraceae and outgroup plastomes (represented by *Nuytsia*). The published plastome of *Nuytsia floribunda* (NC_058869) has been altered (*****) to reflect the gene content found in the plastomes of (**A**) *Sarcophyte*, (**B**) *Thonningia* and *Balanophora* (represented by the plastome of *Thonningia*), and (**C**) *Lophophytum*, *Ombrophytum*, and *Rhopalocnemis* (represented by the plastome of *Rhopalocnemis*). Structural differences in plastome gene order and orientation between *Nuytsia* and Balanophoraceae are highlighted by light blue boxes. Features are numbered from left to right.
